# Isolation and Characterization of *Paracoccus* sp. GSM2 Capable of Degrading Textile Azo Dye Reactive Violet 5

**DOI:** 10.1155/2014/410704

**Published:** 2014-04-23

**Authors:** Mallikarjun C. Bheemaraddi, Santosh Patil, Channappa T. Shivannavar, Subhashchandra M. Gaddad

**Affiliations:** Department of Post Graduate Studies and Research in Microbiology, Gulbarga University, Gulbarga, Karnataka 585106, India

## Abstract

A potential bacterial strain GSM2, capable of degrading an azo dye Reactive Violet 5 as a sole source of carbon, was isolated from textile mill effluent from Solapur, India. The 16S rDNA sequence and phenotypic characteristics indicated an isolated organism as *Paracoccus* sp. GSM2. This strain exhibited complete decolorization of Reactive Violet 5 (100 mg/L) within 16 h, while maximally it could decolorize 800 mg/L of dye within 38 h with 73% decolorization under static condition. For color removal, the most suitable pH and temperature were pH 6.0–9.0 and 25–40°C, respectively. The isolate was able to decolorize more than 70% of five structurally different azo dyes within 38 h. The isolate is salt tolerant as it can bring out more than 90% decolorization up to a salt concentration of 2% (w/v). UV-Visible absorption spectra before and after decolorization suggested that decolorization was due to biodegradation and was further confirmed by FT-IR spectroscopy. Overall results indicate the effectiveness of the strain GSM2 explored for the treatment of textile industry effluents containing various azo dyes. To our knowledge, this could be the first report on biodegradation of Reactive Violet 5 by *Paracoccus* sp. GSM2.

## 1. Introduction

In 1856, the world's first commercially successful synthetic dye, mauveine, was discovered for practical uses. Over 10,000 different dyes with an annual production of over 7 × 10^5^ metric tons worldwide are commercially available [1, 2]. Azo dyes are the diverse group of synthetic organic compounds accounting for the majority of all textile dyestuffs produced and are the most extensively used in a number of industries such as textile dyeing, paper, food, leather, cosmetics, and pharmaceutical industries [3]. The amount of dye lost depends upon the class of dye application, varying from 2% loss while using basic dyes to 50% loss in certain reactive sulfonated dyes, leading to severe contamination of surface and ground waters in the vicinity of dyeing industries [4]. In India, an average mill discharges about 1.5 million liters of contaminated effluent per day, which leads to chronic and acute toxicity [5]. Improper textile dye effluent disposal in aqueous ecosystems leads to the reduction in sunlight penetration which in turn decreases photosynthetic activity, dissolved oxygen concentration, and water quality and depicts acute toxic effects on aquatic flora and fauna, causing severe environmental problems worldwide [6]. They can also cause human health disorders such as nausea, hemorrhage, ulceration of the skin and mucous membranes, and severe damage to kidneys, the reproductive system, liver, brain, and central nervous system [7]. In addition, azo dyes also have an adverse impact in terms of total organic carbon (TOC), biological oxygen demand (BOD), and chemical oxygen demand (COD) [8]. Many synthetic azo dyes and their metabolites are toxic, carcinogenic, and mutagenic [9]. Therefore, the treatment of industrial effluents containing azo dyes and their metabolites is necessary prior to their final discharge to the environment.

Various physicochemical methods like adsorption, chemical precipitation and flocculation, photolysis, chemical oxidation and reduction, electrochemical treatment, and ion pair extraction have been used for the removal of dyes from wastewater [10]. The major drawbacks of these methods have been largely due to the high cost, low efficiency, limited versatility, interference by other wastewater constituents, and the handling of the waste generated [11]. Conversely, biological processes provide an alternative to existing technologies because they are more cost-effective, environmental friendly and do not produce large quantities of sludge. Many microorganisms belonging to the different taxonomic groups of bacteria, fungi, actinomycetes, and algae have been reported for their ability to decolorize azo dyes [12]. Pure fungal cultures have been used to develop bioprocesses for the mineralization of azo dyes, but the long growth cycle and moderate decolorization rate limit the performance of fungal decolorization system [13]. In contrast, bacterial decolorization is normally faster. Bacteria capable of dye decolorization/biodegradation either in pure cultures or in consortia have been reported [11, 14–17]. However, comprehensive solutions for sulfonated azo dyes removal are far from reality, which calls for continued search for new organisms and technologies.

This study aimed to isolate and characterize an efficient bacterial strain, which exhibited the remarkable ability to degrade Reactive Violet 5 as a sole source of carbon. Various physicochemical parameters have been optimized for efficient dye decolorization. The dye degraded products were characterized by ultraviolet-visible (UV-Vis) and Fourier transformed infrared spectroscopy (FT-IR) techniques. Very few reports are available on Reactive Violet 5 degradation. After survey of the literature, this could be the first report on biodegradation of Reactive Violet 5 by* Paracoccus* sp. GSM2.

## 2. Materials and Methods

### 2.1. Dyes and Chemicals

**Figure 1:**
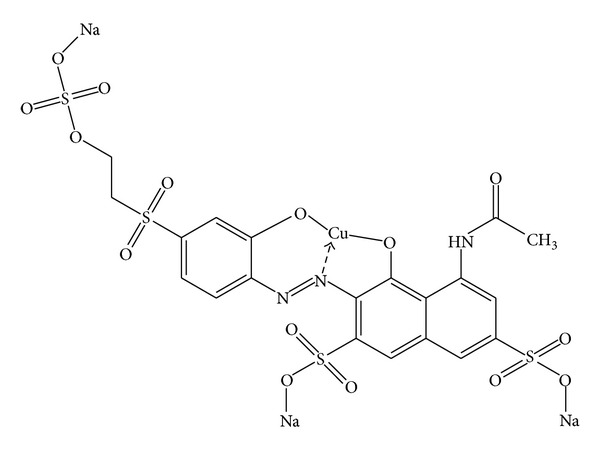
Structure of Reactive Violet 5.

Six textile azo dyes Reactive Violet 5, Reactive Red 2, Reactive Orange 16, Reactive Blue 4, Reactive Black 5, and Reactive Green 19 A were generous gifts from Colors India Inc. Pvt. Ltd., Ahmedabad, India. All these dyes were of industrial grade and are widely used in textile industries. Reactive Violet 5 was used as a model azo dye in this study (Figure 1). All required chemicals were obtained from S.D. Fine chemicals (India) and Sigma-Aldrich, (USA). Biochemical and physiological test kits were obtained from Hi-Media, India. All chemicals used during the study were of analytical grade.

### 2.2. Culture Medium

The mineral salts medium (MSM) was prepared as per Brilon et al. [18] with some modifications. The MSM consisted of the following constituents (g/L): Na_2_HPO_4_·2H_2_O (12.0), KH_2_PO_4_ (2.0), NH_4_NO_3_ (0.50), MgCl_2_·6H_2_O (0.10), Ca(NO_3_)_2_·4H_2_O (0.050), and FeCl_2_·4H_2_O (0.0075) with 10 mL of trace element solution per liter. The trace element solution was prepared as follows (mg/L): ZnSO_4_·7H_2_O (10.0), MnCl_2_·4H_2_O (3.0), CoCl_2_·6H_2_O (1.0), NiCl_2_·6H_2_O (2.0), Na_2_MoO_4_·2H_2_O (3.0), H_3_BO_3_ (30.0), and CuCl_2_·2H_2_O (1.0). Further, MSM was blended with different concentrations of Reactive Violet 5 and used throughout the study as a test medium and uninoculated flasks were also incubated as control. The final pH of the medium was adjusted to 7.0 ± 0.2. The MSM with agar (1.9% w/v) was used for isolation and maintenance of pure culture. The media were sterilized at 121°C for 20 min before use.

### 2.3. Screening, Isolation, and Identification of Dye Decolorizing Bacteria

Textile mill effluent collected from Solapur, India, was brought to the laboratory for isolation of dye degrading bacteria. 10 mL of sample was added to 100 mL of MSM broth containing Reactive Violet 5 (100 mg/L) as a sole source of carbon and incubated at 30°C for 15 days under static as well as shaking conditions. The flasks were checked for change in initial color and turbidity. Then 10 mL of culture broth from the decolorized culture flask was transferred to 100 mL of fresh MSM broth containing Reactive Violet 5 and incubated for one week under static condition. 0.5 mL of decolorized culture was taken out and spread over the agar plates of MSM containing dye and incubated at 30°C until prominent dye degrading bacterial colonies appeared. Further the prominent colonies were streaked onto the MSM agar plates amended with dye and 0.1% (w/v) yeast extract. The obtained colonies formed were screened out and further were checked for the purity by streaking twice on agar medium. Finally, purified cultures were individually tested for their dye degrading capabilities in MSM under static condition. The potential isolate was preserved at −20°C in 15% (w/v) glycerol and used for further investigation. The potential isolate was selected and preliminarily characterized based on its morphological and biochemical properties [19]. Furthermore, various sugar utilization tests were performed using HiCarbo kit (Hi-Media, India).

#### 2.3.1. 16S rDNA Sequencing and Analysis

The 16S rDNA fragment was amplified and from the pure genomic DNA of isolated bacterial strain was sequenced at Royal Life Sciences Pvt. Ltd., Hyderabad, India. The genomic DNA was extracted using QIAGEN bacteria DNA purification kit according to manufacturer's instructions. The universal primers, namely, a forward primer, Eub27F (5′–3′: AGA GTT TGA TCC TGG CTC AG), and a reverse primer, Eub1492R (5′–3′: ACG GCT ACC TTG TTA CGA CTT), were used to amplify bacterial 16S rDNAs by PCR which yielded a product of approximately 1500 bp. After an initial denaturation at 95°C for 10 min, the DNA was amplified during 25 cycles of 95°C for 1 min, 55°C for 1 min, and 72°C for 1.5 min and the final extension (72°C) time was 10 min. Then the purified PCR products were run on ABI-PRISM automated sequencer (ABI-3730 DNA Analyzer). A resultant of 1311 bases was compared with nine closely related taxa of the isolate, retrieved from the GenBank database using BLAST (blastn) program on the NCBI website (http://www.ncbi.nlm.nih.gov). The alignment of the sequences was done using CLUSTALW program V 1.6 at European bioinformatics site (http://www.ebi.ac.uk/Tools/msa/). The sequence was refined manually after crosschecking with the raw data to remove ambiguities and submitted to GenBank. To see the phylogenetic position of bacterial isolate evolutionary history was inferred using the neighbor-joining method [20]. The optimal tree with the sum of branch length = 0.28801765 is shown. The percentage of replicate trees in which the associated taxa clustered together in the bootstrap test (1000 replicates) is shown next to the branches [21]. The phylogenetic tree was linearized assuming equal evolutionary rates in all lineages [22]. The clock calibration to convert distance to time was 0.02 (time/node height). The tree is drawn to scale, with branch lengths in the same units as those of the evolutionary distances used to infer the phylogenetic tree. The evolutionary distances were computed using the maximum composite likelihood method [23] and are in the units of the number of base substitutions per site. Codon positions included were 1st + 2nd + 3rd + Noncoding. All positions containing gaps and missing data were eliminated. Evolutionary analyses were conducted in MEGA5 software [24].

### 2.4. Decolorization Experiment

The dye decolorization experiments were performed in 250 mL Erlenmeyer flasks containing 100 mL of sterilized MSM broth supplemented with yeast extract (0.1% w/v) and Reactive Violet 5 (100 mg/L). We recorded complete decolorization of Reactive Violet 5 in MSM with yeast extract within 16 h as compared to 56 h without yeast extract under the static condition (data not shown). Reports suggest that the inclusion of yeast extract was found to be most effective supplement for growth of azo dye degrading bacteria as well as increasing the dye decolorization efficiency [13, 25]. Therefore all further decolorization experiments were performed using MSM broth supplemented with 0.1% (w/v) yeast extract as a cosubstrate. The flasks were inoculated with 5 mL of cultures broth in test and uninoculated controls were used to compare abiotic color loss during the decolorization studies. The flasks were incubated at 30°C under static as well as shaking (120 rpm) conditions till the decolorization was completed. The 5 mL of cultures was withdrawn at different intervals for color measurement. The supernatant was collected by centrifuging at 10,000 rpm for 15 min. Decolorization was monitored spectrophotometrically by measuring absorbance of culture supernatant at 558 nm. Growth of bacteria in dye containing medium was determined spectrophotometrically. The cell pellet obtained upon centrifugation of 5 mL culture was resuspended in 5 mL distilled water and its absorbance was studied at 660 nm. The percentage of decolorization was calculated as mentioned by R. Dave and H. Dave [26]:

(1) $$\begin{array}{c} \text{Decolorization}\;\left(\%\right)\;=\;\frac{I\;-\;F}{I}\;\times \;100, \end{array}$$

where *I* = initial absorbance and* F* = absorbance of decolorized sample.

### 2.5. Optimization of Physicochemical Parameters

The decolorization efficiency of* Paracoccus* sp. GSM2 on Reactive Violet 5 was studied at different pH (4–10) and temperatures values (20–50°C). The obtained optimum pH 7.0 and temperature at 30°C were selected to study the decolorization activity under various physicochemical factors such as initial dye concentration (100–800 mg/L), salt concentration (1–6%), and yeast extract concentration (0.1–2.0 g/L). Further, the decolorization of various azo dyes was studied by incubating MSM containing respective dye with bacterial strain GSM2 under static condition.

### 2.6. Decolorization and Biodegradation Studies

The Reactive Violet 5 degraded products formed during biodegradation after 16 h of incubation under static condition were studied by following the change in the UV-Vis spectra (200 to 800 nm) using a UV-Vis spectrophotometer (Systronics, AU-2700). To know that the decolorization was due to biodegradation of Reactive Violet 5 was confirmed by FT-IR by analyzing dye degraded products in the decolorized medium. After complete decolorization, culture medium was centrifuged at 10,000 rpm for 15 min to remove the suspended particles. The supernatant was once again centrifuged to ensure the supernatant was free of bacterial cells and was used for extraction of metabolites using an equal volume of ethyl acetate. The extracts were dried over anhydrous Na_2_SO_4_ and concentrated in a rotary evaporator. The crystals obtained were dissolved in a small volume of high performance liquid chromatography (HPLC) grade methanol and the same sample was used for FT-IR analysis. The FT-IR analysis of extracted metabolites was done using Fischer Scientific (Nicolet, iH5) Spectrophotometer and compared with control dye in the IR region of 550–4000 cm^−1^ with 32 scan speed.

## 3. Results and Discussion

### 3.1. Isolation, Screening, and Identification

**Figure 2:**
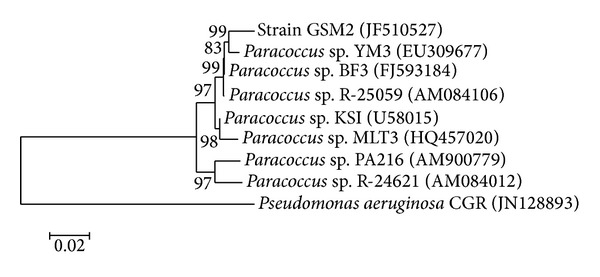
Phylogenetic tree of* Paracoccus* sp. GSM2 based on 16S rDNA analysis.

A total of seven morphologically distinct colonies were observed on the MSM agar plates (data not shown). Amongst positive strains subjected to screening, the potential bacterial strain GSM2 showed a rapid and complete decolorization of Reactive Violet 5 within 16 h under static condition and was selected for further study. The selected strain was gram negative, nonspore former, and nonmotile coccoid. Its colony was white to light yellow, round hunch, and slick. The potential strain was identified as* Paracoccus* sp. GSM2 on the basis of 16S rDNA gene sequence and biochemical characteristics (Table 1). The 16S rDNA sequence (1311 base pairs) was deposited in GenBank with accession number JF510527. Its 16S rDNA sequence did not show any similarity to known azo dye degrader and had the greatest similarity to members of* Paracoccus* sp. group. The phylogenetic relationship between the* Paracoccus* sp. GSM2 and other related microorganisms using MEGA5 software can be depicted from Figure 2.* Pseudomonas aeruginosa* CGR has been taken as out-group and the numbers shown in parentheses are accession numbers of different species.

The homology assay result indicated that the* Paracoccus* sp. GSM2 in phylogenetic branch showed maximum similarity (99%) to* Paracoccus* sp. YM3 which is already known for degradation of carbofuran [27]. Few reports are published on degradation of polycyclic aromatic hydrocarbons by* Paracoccus* sp. [28, 29]. To our knowledge, this could be the first report on biodegradation of textile azo dye Reactive Violet 5 by* Paracoccus* sp. GSM2.

### 3.2. Effect of Static and Shaking Conditions

**Figure 3:**
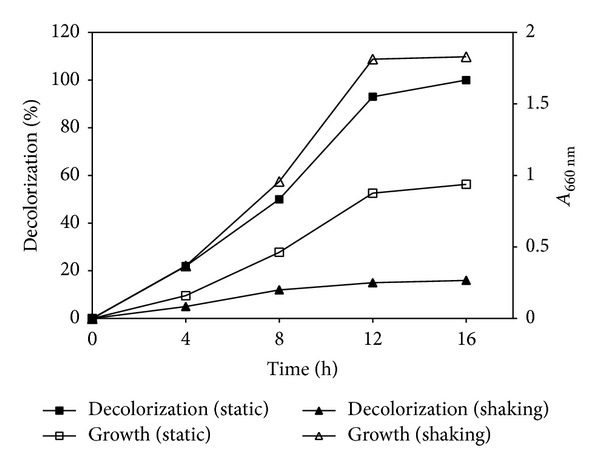
Decolorization of Reactive Violet 5 by* Paracoccus* sp. GSM2 in MSM under static and shaking condition (120 rpm) at 30°C.

*Paracoccus* sp. GSM2 showed that 100% decolorization of added Reactive Violet 5 (100 mg/L) within 16 h under static condition when compared to only 16% decolorization was observed under shaking condition, while the growth of bacterium was greater under shaking condition as compared to static condition (Figure 3). To confirm whether this decolorization was due to microbial action or due to change in pH, the change in pH was recorded, which was in the range of 6.0–7.5 at static condition, thus confirming that the biodegradation of dye was due to microbial action. Under aerobic conditions azo dyes are generally resistant to attack by bacteria [30]. Similar findings were reported by other researchers [14, 15]. During dye decolorization in shaking environment electrons released by oxidation of electron donors are preferentially utilized to reduce free oxygen rather than azo dyes [31]. Hence, in this study static conditions were maintained in the following experiments.

### 3.3. Effect of pH

**Figure 4:**
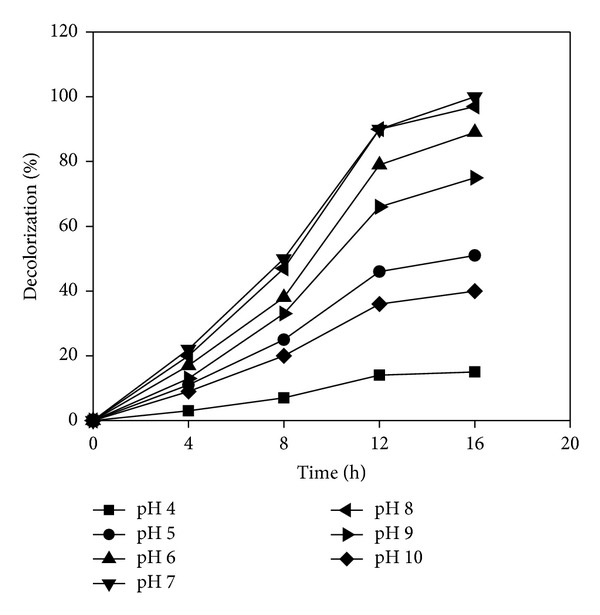
Effect of pH on decolorization of Reactive Violet 5.

The effect of pH on decolorization of Reactive Violet 5 by* Paracoccus* sp. GSM2 was determined over a wide range of pH 4.0 to 10.0 with an interval of pH 1. The isolate showed the maximum of 100% decolorization at pH 7.0 at 30°C. Following the increases from either side of neutral pH, the percentage of decolorization decreased steadily from 97% to 40% on the alkaline side while steep decline in percent decolorization from 89% to less than 15% on acidic side was found. More than 88% of decolorization was observed in a wide range of pH 6.0 to 8.0 (Figure 4). Similar optimum pH was observed in the decolorization of the same dye Reactive Violet 5 by bacterial consortium SB4 [14]. Chan and Kuo [32] reported that the neutral pH would be more favorable for decolorization of the azo dyes and is suitable for industrial applications.

### 3.4. Effect of Temperature

**Figure 5:**
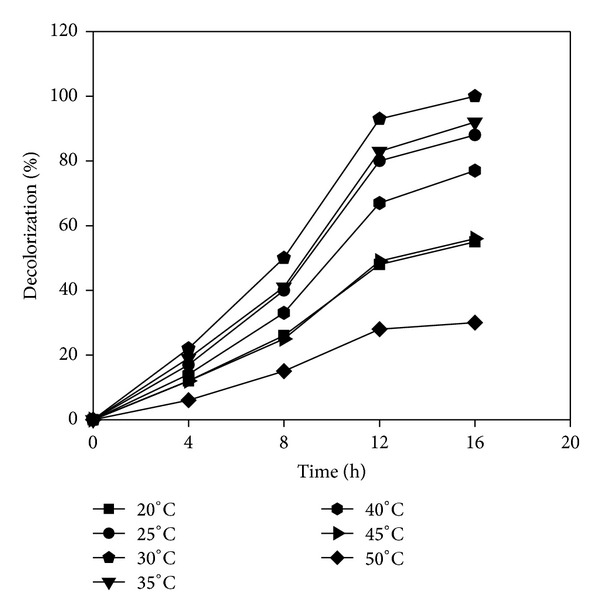
Effect of temperature on decolorization of Reactive Violet 5.

Similarly in the temperature optimization study, the dye decolorization activity of* Paracoccus* sp. GSM2 was found to increase with increase in incubation temperature from 20 to 30°C. Further increase in temperature, decolorization was decreased by 23% and 44% at 40°C and 45°C, respectively, and almost no activity was found at 50°C (Figure 5). This might be attributed to the adverse effect of high temperature on enzyme activities [33]. Tamboli et al. [16] also found the decrease in dye decolorization efficiency of* Sphingobacterium* sp. ATM for color removal beyond 30°C, which may be due to the thermal inactivation of the decolorizing enzymes.

### 3.5. Effect of Initial Dye Concentration

**Figure 6:**
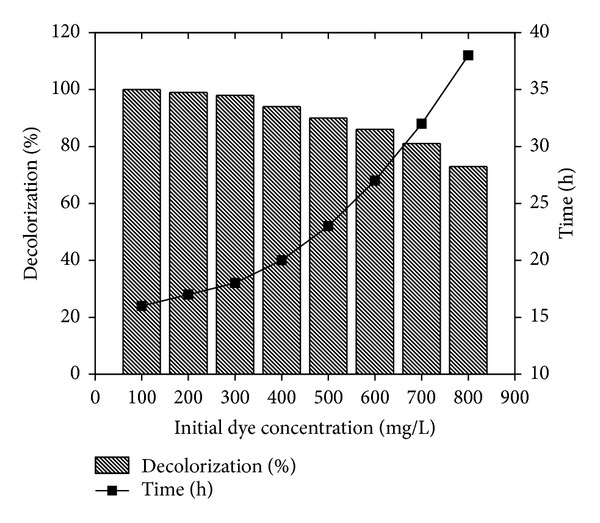
Effect of initial dye concentration on decolorization of Reactive Violet 5.

The decolorization performance of Reactive Violet 5 by the* Paracoccus* sp. GSM2 was studied by increasing initial dye concentration (100–800 mg/L). We observed that the percentage of decolorization was decreased slowly with increasing dye concentration (Figure 6). It could effectively decolorize up to 100 mg/L of Reactive Violet 5 (100%) within 16 h and is decreased to 63%, when dye concentration increased to 800 mg/L and decolorization time increases from 16 h to 38 h, respectively. Lower percentage of decolorization and enhanced time period at high dye concentration may be attributed to the presence of four sulfonic acid groups on Reactive Violet 5 which acts as detergent exerting inhibitory effect on growth of* Paracoccus* sp. GSM2 [14].

### 3.6. Effect of Salt Concentration

**Figure 7:**
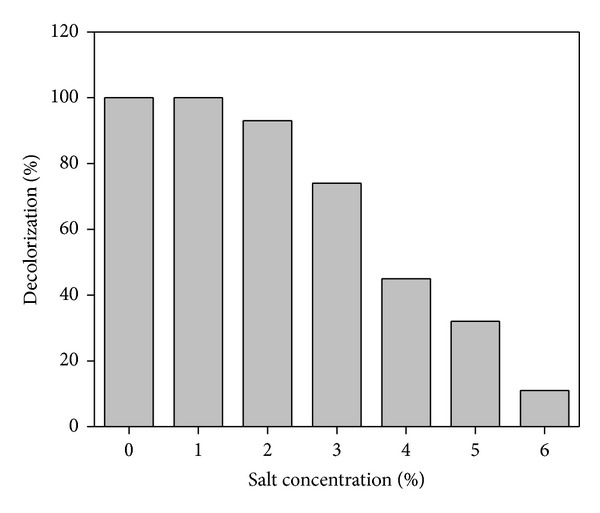
Effect of salt concentration on decolorization of Reactive Violet 5.

Textile industry effluents generally contain chloride salts of sodium and potassium which are most widely used for salting out of dyes and discharged along with unused dyes. Hence, the present investigation was undertaken to study the effect of salt concentration (1–6% w/v) on decolorization of Reactive Violet 5 by the strain GSM2. The organism showed satisfactory decolorization up to maximum of 2% salt concentration in MSM under optimum conditions after 16 h of incubation (Figure 7). Previously De Baere et al. [34] have stated that sodium concentration higher than 3 g/L can cause inhibition of most of the bacterial metabolism. But, contrary to the above statement, we could notice 74% and 45% of decolorization at 3% and 4% of salt concentration, respectively. Which agree with the earlier report [14]. Negligible activity was observed when 6% of salt concentration was employed into the medium. This may be attributed to the inhibition of bacteria at high salt concentration due to plasmolysis or loss of activity of cells [35].

### 3.7. Effect of Different Concentrations of Yeast Extract

**Figure 8:**
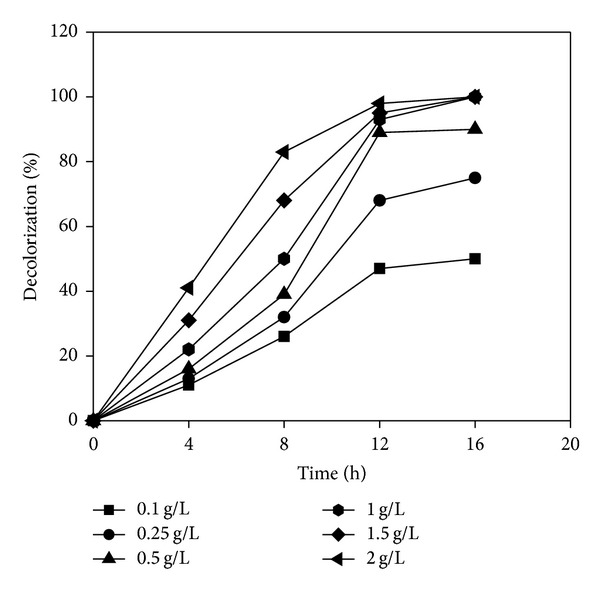
Effect of yeast extract concentration on decolorization of Reactive Violet 5.

*Paracoccus* sp. GSM2 was able to degrade Reactive Violet 5 (100 mg/L) efficiently in the presence of yeast extract as a cosubstrate. Among all other nitrogen sources, only yeast extract that served as better nitrogen source for decolorization of Reactive Violet 5 within less time was selected for further experiments (data not shown). Effect of different concentrations of yeast extract (0.1–2.0 g/L) in MSM on the decolorization efficacy of GSM2 was evaluated (Figure 8). Addition of 1 g/L of yeast extract enhanced the decolorization activity and complete decolorization of Reactive Violet 5 was recorded within 16 h. Further increase in yeast extract concentration has no effect on decolorization activity. Thus, to make the process economical 1 g/L of yeast extract concentration was found to be optimum. Similar results were reported by Jain et al. [14] where their findings proposed that yeast extract was essential for regeneration of NADH which acts as an electron donor in azo bond reduction.

### 3.8. Decolorization of Structurally Different Azo Dyes

Structurally different azo dyes were most widely used in textile processing industries, and, therefore, the effluents from the industry are markedly diverse in composition [36]. Thus,* Paracoccus* sp. GSM2 was tested for its ability to decolorize five other structurally different azo dyes such as Reactive Red 2, Reactive Orange 16, Reactive Black 5, Reactive Blue 4, and Reactive Green 19 A. The organism effectively decolorized all structurally different azo dyes within 38 h (Table 2). The efficiency was 100% for Reactive Red 2, followed by 99% for Reactive Orange 16, 92% for Reactive Blue 4, 82% for Reactive Black 5, and 73% for Reactive Green 19 A. We presume that decolorization of structurally different azo dyes by* Paracoccus* sp. GSM2 within 38 h might be the first. This variation in the decolorization of different dyes might be attributed to the structural differences, high molecular weight, and presence of inhibitory groups like -NO_2_ and -SO_3_Na in the dyes [36]. The present study confirms the ability of strain GSM2 to decolorize different azo dyes with decolorization efficiency of more than 70%. Thus, the strain GSM2 could be successfully employed for the treatment of textile industry effluents containing various azo dyes.

### 3.9. Decolorization and Biodegradation Studies

**Figure 9:**
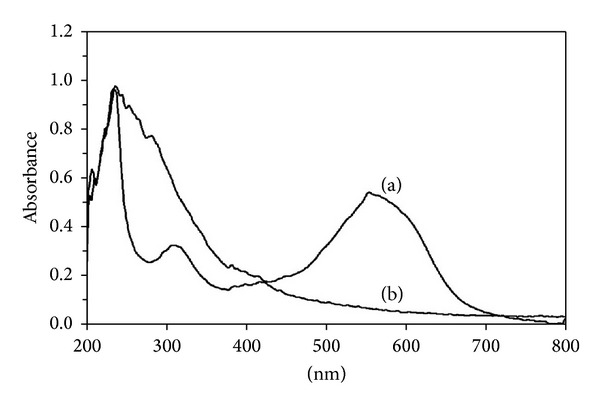
UV-Vis spectra of Reactive Violet 5 before and after decolorization by* Paracoccus *sp. GSM2 (a, 0 hour; b, 16 hours).

**Figure 10:**
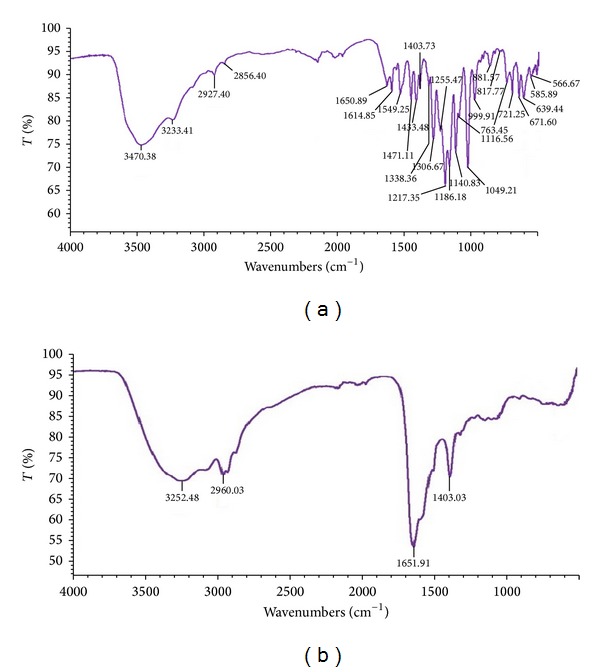
FT-IR spectra of (a) control Reactive Violet 5 and (b) its degradation metabolites.

To disclose the possible mechanism of dye decolorization, we also analyzed the degraded products of Reactive Violet 5 by UV-Vis and FT-IR techniques. UV-Vis absorbance of 200–800 nm of Reactive Violet 5 in MSM showed single peak in visible region at 558 nm (*λ*_max⁡_) and two intense peaks in UV region near 250 and 325 nm, respectively, correspond to phenyl and naphthyl rings of Reactive Violet 5 (Figure 9) [37]. During decolorization azo bond in Reactive Violet 5 was broken down and peak at 558 nm continuously decreased and completely disappeared within 16 h, without any shift in *λ*_max⁡_. Similar observations have been recorded by Jain et al. [14]. According to Asad et al. [12] decolorization of dyes by bacteria could be due to adsorption by microbial cells or to biodegradation. In the case of adsorption, the UV-Vis absorption peaks decrease approximately in proportion to each other, whereas, in biodegradation, either the major visible light absorbance peak disappears completely or a new peak appears. The observation of cell mass showed that* Paracoccus* sp. GSM2 retained their natural color after decolorization of Reactive Violet 5. FT-IR spectrum of control dye with metabolites extracted after complete decolorization (16 h) clearly indicated the biodegradation of the parent dye compound by* Paracoccus* sp. GSM2 (Figure 10). FT-IR analysis of control and decolorized samples showed significant differences in specific peaks of Reactive Violet 5 fingerprint region (550–4000 cm^−1^). FT-IR spectra of control Reactive Violet 5 show specific peaks for multisubstituted benzene ring, where peaks at 1140.83, 1338.36, 1186.18, and 1549.25 cm^−1^ corresponded to two SO_3_H groups, symmetric SO_2_, and –N=N– (azo bond), whereas parasubstituted azo benzene showed bands near 1433.48 cm^−1^. Reactive Violet 5 is a metal containing textile azo dye where carbonic ion is bonded with a central copper metal ion giving rise to asymmetrical stretching which was observed near 1614.85 cm^−1^ [38]. Peak at 1650.89 cm^−1^ represents the primary amide of the parent structure of Reactive Violet 5. The FT-IR spectra of 16 h extracted metabolites of degraded Reactive Violet 5 showed peaks at 1651.91 and 3252.48 cm^−1^ which indicates the production of primary amine and secondary amide, respectively, during biodegradation of Reactive Violet 5. Absence of peaks at 671, 721, 763, and 817 cm^−1^ indicates the breakdown of benzene ring or the loss of aromatic nature of the compound. Jain et al. [14] reported similar kind of benzene ring fission in the same dye Reactive Violet 5 by bacterial consortium SB4. Correspondingly, breakdown of azo bond was confirmed by the absence of spectral peak at 1549.25 cm^−1^, while the absence of the peaks around 1300 and 1165–1150 cm^−1^ clearly indicates the degradation of S=O bonds. Peaks at 2927.40 and 2960.03 cm^−1^ in control Reactive Violet 5 and degraded metabolites, respectively, show the asymmetrical stretching of C–H in CH_3_. Similar kind of asymmetrical stretching of peak at 2856 cm^−1^ in control Reactive Violet 5 shows that the asymmetrical stretching for C–H stretching was observed in degradation of the same dye Reactive Violet 5 [39]. On the basis of above results, it can be concluded that* Paracoccus* sp. GSM2 has ability to mineralize Reactive Violet 5 completely.

## 4. Conclusion

The present study showed that a bacterial strain* Paracoccus* sp. GSM2 is capable of degrading Reactive Violet 5 as a sole source of carbon with minimal nutritional requirements under static condition. The potential of this strain has ability to decolorize Reactive Violet 5 in a wide range of pH, temperature, salt, and initial dye concentrations, which is significant for its commercial application. The FT-IR results showed complete loss of the aromatic nature of the dye Reactive Violet 5 by* Paracoccus* sp. GSM2. Furthermore, strain GSM2 had the ability to decolorize five other structurally different azo dyes indicating its field applicability in the treatment of textile effluents. Therefore,* Paracoccus* sp. GSM2 is the highly promising bacterium that can be used for the treatment of textile industry effluents containing various reactive azo dyes.

## Figures and Tables

**Table 1:** Biochemical and sugar utilization tests of bacterial strain GSM2.

Characteristics	Bacterial strain GSM2
Catalase	−
Oxidase	+
Urease	+
Citrate utilization	−
Nitrate reduction	+
Glucose	+
Adonitol	+
Lactose	−
Sorbitol	+
Esculin hydrolysis	−
Xylose	−
Maltose	+
Fructose	+
Galactose	−
Raffinose	−
Trehalose	+
Melibiose	−
Sucrose	+
L-Arabinose	+
Mannose	+
Inulin	+
Sodium gluconate	+
Glycerol	+
Salicin	+
Dulcitol	+
Inositol	+
Mannitol	[+]
Arabitol	+
Erythritol	[+]
*α*-Methyl-D-glucoside	+
Rhamnose	+
Cellobiose	+
Melezitose	+
*α*-Methyl-D-mannoside	−
Xylitol	−
ONPG	−
D-Arabinose	+
Malonate utilization	+
Sorbose	−

+: positive, [+]: weakly positive, and −: negative.

**Table 2:** Decolorization of structurally different azo dyes by *Paracoccus s*p. GSM2.

Reactive dyes	*λ*_ max_ (nm)	% Decolorization	Time (hours)
Reactive Red 2	538	100	12
Reactive Orange 16	416	99	18
Reactive Blue 4	595	92	22
Reactive Black 5	597	82	30
Reactive Green 19A	540	73	38
